# On the Powers of Strychnia in the Cure of Chronic Bronchitis

**Published:** 1847-04

**Authors:** P. Henry Clarke

**Affiliations:** Port Washington, W. T.


ARTICLE II.

### *On the powers of Strychnine in the cure of Chronic Bronchitis.*

By Dr. P. Henry Clarke, of Port Washington, W. T.

In the treatment of no disease, or class of diseases, have I experienced more difficulty than in bronchial affections. And the utter failure of the practice, recommended by authors, proves conclusively the absolute necessity of more attention being bestowed upon this subject. With this view, I offer to the consideration of the profession, a few remarks, not however preferring any claim to originality, but rather wishing to impress upon the minds of those afflicted, the actual necessity of making due exertions for relief, notwithstanding such disease may, perhaps, be pronounced incurable.

Strychnine was discovered by Mons. Pellitier and Caventou, in the year 1818, and named by them Vauqueline, in honor of that distinguished chemist. After which they changed the name to Strychnine. It is so intensely bitter that it is said to give a decided taste to 600,000 parts of water by weight, and is but sparingly soluble in alcohol. It is one of the most virulent poisons; and, perhaps, inferior to none, except the highly concentrated prussic acid. Majendie killed a dog, by the administration of one eighth of a grain. Its effects are to produce tetanus, and consequent immobility of the thorax, asphyxia and death. The curative effects of Strychnine in cases of Paralysis, both general and partial, as in hemiplegia and paraplegia, also in tetanus, obstinate cases of amenorrhœa, in chronic diarrhœa without pain and with thin serous discharges, which produce exhaustion, and in nearly all the various diseases to which the eye is subject, are too well known to require an insertion here.

My method of administering it is principally in powder, suspended in mucilage as a vehicle, or by making it into pills, preferring either to the tincture, owing to the insolubility of it in alcohol. In anhydrous alcohol it is perfectly insoluble, consequently no tincture can be prepared that will give an equal strength.

Having been afflicted severely for quite a number of years with Bronchitis, and finding no medicines which gave me relief, I was induced to try the effect of strychnine, which resulted in a perfect cure. My symptoms, when I commenced using it, were emaciation, night-sweats, and continued mucous expectoration, attended with cough, at times very severe, after which the muscles of the larynx were so completely relaxed, that I could not utter a sound above a whisper, but unattended with pain. I commenced the use of the strychnine, as advised, by taking one-twentieth of a grain, suspended in mucilage, three times in a day and increased the dose every third day until I took one-fifth of a grain. I used the remedy about four weeks, and have never experienced any difficulty since. I was much astonished at its results, and more especially at the effects it produced upon the contractility of the muscles of the larynx, as well as upon the muscles of the extremities.

Case II.—A. B. S., an attorney, after delivering an address, and exercising unusally hard the organs of speech by talking very loud, in returning home was caught in a shower, and drenched to the skin. Immediately after he was attacked with Acute Bronchitis. I knew nothing in regard to his treatment, but it resulted in Chronic Bronchitis, and he was unable to speak, for over three years, above a whisper. I prevailed upon him to make use of strychnine, and gave it in pills made with flour, and ext. liquorice, and one-thirteenth of a grain of strychnine, increased until he took one-eighth of a grain; which amount he continued to take for nearly two months. He now experiences no difficulty in speaking, and thinks he has obtained a complete cure.

Case III.—E. W. L, Aét. 52, an itinerant preacher, was by degrees entirely deprived of speech, and remained thus for twelve or fifteen years. He had some cough and expectoration, slightly tinged with blood. He was, by my recommendation, last spring, induced to make use of the strychnine. Two or three months since I received a note from him stating that he was not perfectly cured but sufficiently so to be able to speak in public, if he used moderation. After speaking he felt still an oppressive weakness in his chest, and slight tracheal irritation, but not sufficient to produce cough. He was directed to continue the use of strychnine as before. I have not heard from him since.

Case IV.—A lady afflicted with occasional loss of speech, with neither cough or expectoration, but extreme emaciation, and had as she expressed it, a continued "tickling and hacking." She was ordered to take strychnine. This I gave in tincture, six grs. to the fluid ounce of diluted alcohol. She commenced with three drops three times a day, and increased as in the other cases. This was attended with the most decided success.

Where there is a local determination of blood to the head it is necessary to deplete until that is removed, before using the strychnine. Morphine, to a certain extent, is an antidote to strychnine. Lembert introduced three grains of strychnine under the skin of a dog on one side of the spine, and six grains of morphine on the other side, without any visible effects following it; either of which alone would have caused death.

I have seen it used in one well marked case of Phthisis Pulmonalis, but its effects could not be ascertained to my entire satisfaction, as the patient at the same time freely used Wistar's Balsam of Wild Cherry. He recovered rapidly, and was, at the last report that he made me, almost, if not entirely, well. Over two years had elapsed since he used the strychnine. From the effects which it made upon the system, I judged that his recovery was attributable to that remedy alone. His case was hereditary. His father, brother and sister died with that complaint; every one had given him up as incurable. I have never seen any ill effects result from the use of Strychnine, unless perhaps in one case. But so virulent a poison is it, that the utmost precaution is necessary in its administration to prevent accident. The case to which I refer above, was one of complete paralysis. I used the Tinc. in a dose of six drops three times a day, and at the same time used drastic cathartics and venesection. He recovered entirely from the paralysis. In about five months he was taken with an abscess in the thigh, and gangrene immediately followed. I should have been happy to have noticed the appearance of the subsequent disease, but was not aware that he had been sick, until I heard of his demise. Whether the use of the strychnine had any effect in the production of the subsequent disease, or not, I cannot say, but suppose if so it would have developed itself sooner. But in regard to strychnine as a remedy in Bronchial affections, and even in the forming stages of Phthisis Pulmonalis, I have the most implicit confidence in it, and think that the time is not far distant when Pulmonary Consumption shall be stripped of its manifold terrors, by the sanitary influence of this powerful remedial agent.

